# Assessment of boron-containing compounds and oleoylethanolamide supplementation on the recovery trend in patients with COVID-19: A structured summary of a study protocol for a randomized controlled trial

**DOI:** 10.1186/s13063-020-04820-2

**Published:** 2020-10-27

**Authors:** Helda Tutunchi, Majid Mobasseri, Samira Pourmoradian, Hamid Soleimanzadeh, Behnam Kafil, Neda Akbari, Alireza Monshikarimi, Alireza Ostadrahimi

**Affiliations:** 1 Department of Clinical Nutrition, Nutrition Research Center, School of Nutrition & Food Sciences, Tabriz University of Medical Sciences, Tabriz, 5166614711 Iran; 2 Endocrine Research Center, Tabriz University of Medical Sciences, Tabriz, Iran; 3 Nutrition Research Center, Tabriz University of Medical Sciences, Tabriz, Iran; 4 Department of Applied Chemistry, Faculty of Chemistry, University of Tabriz, Tabriz, Iran

**Keywords:** COVID-19, Randomised controlled trial, Protocol, Oleoylethanolamide, Boron-containing compounds, Complete blood count, Erythrocyte sedimentation rate, C-reactive protein

## Abstract

**Objectives:**

In this study, we investigate the effect of boron-containing compounds and oleoylethanolamide supplementation on the recovery trend in patients with COVID-19.

**Trial design:**

The current study is a single-center, randomized, double-blind, placebo-controlled clinical trial with parallel groups.

**Participants:**

The inclusion criteria include male and female patients≥18 years of age, with a confirmed diagnosis of SARS-CoV-2 infection via polymerase chain reaction (PCR) and/or antibody test and with written informed consent to participate in this trial. The exclusion criteria include regular use of any other supplement, severe and critical COVID-19 pneumonia, pregnancy and breastfeeding. This study is being conducted at Imam Reza Hospital, Tabriz University of Medical Sciences, Tabriz, Iran.

**Intervention and comparator:**

Patients are randomly assigned to four groups. The first group (A) will take one capsule containing 5 mg of boron compounds twice a day for two weeks. The second group (B) will take one capsule containing 200 mg oleoylethanolamide twice a day for two weeks. The third group (C) will take one capsule containing 5 mg boron compounds with 200 mg oleoylethanolamide twice a day for two weeks, and the fourth group (D) does not receive any additional treatment other than routine treatments. Boron-containing compounds and oleoylethanolamide capsules will be synthesized at Nutrition Research Center of Tabriz University of Medical Sciences.

**Main outcomes:**

The primary end point of this study is to investigate the recovery rate of clinical symptoms, including fever, dry cough, and fatigue, as well as preclinical features, including complete blood count (CBC), the erythrocyte sedimentation rate (ESR), C-reactive protein (CRP) profiles within two weeks of randomization.

**Randomisation:**

Patients are randomized into four equal groups in a parallel design (allocation ratio 1:1). A randomized block procedure is used to divide subjects into one of four treatment blocks (A, B, C, and D) by a computer-generated allocation schedule.

**Blinding (masking):**

The participants and investigators (enrolling, assessing, and analyzing) are blinded to the intervention assignments until the end of the study and data analysis.

**Numbers to be randomised (sample size):**

The calculated total sample size is 40 patients, with 10 patients in each group.

**Trial Status:**

The protocol is Version 1.0, May 17, 2020. Recruitment began May 19, 2020, and is anticipated to be completed by October 19, 2020.

**Trial registration:**

This clinical trial has been registered by the title of “*Assessment of boron-containing compounds and oleoylethanolamide supplementation on the recovery trend in Patients with COVID-19: A double-blind randomized placebo-controlled clinical trial*” in the Iranian Registry of Clinical Trials (IRCT). The registration number is “IRCT20090609002017N35”, https://www.irct.ir/trial/48058. The registration date is 17 May 2020.

**Full protocol:**

The full protocol is attached as an additional file, accessible from the Trials website (Additional file 1). In the interest in expediting dissemination of this material, the familiar formatting has been eliminated; this Letter serves as a summary of the key elements of the full protocol.

**Supplementary information:**

**Supplementary information** accompanies this paper at 10.1186/s13063-020-04820-2.

## Supplementary information

**Additional file 1.**

## Data Availability

The corresponding author has access to the final dataset of the trial, and the data will be available on reasonable request (Contact: Ostadrahimi@tbzmed.ac.ir).

