# Supplementary material for: Assessment of boron-containing compounds and oleoylethanolamide supplementation on the recovery trend in patients with COVID-19: A structured summary of a study protocol for a randomized controlled trial
Source: Trials. 2020 Oct 27;21:890. doi: 10.1186/s13063-020-04820-2 (PMC7588946; doi:10.1186/s13063-020-04820-2)
Supplement: Supplementary file 1 — Additional file 1. [file 13063_2020_4820_MOESM1_ESM.docx]

**Full Protocol**

**Assessment of boron-containing compounds and oleoylethanolamide supplementation on the recovery trend in patients with COVID-19: A structured summary of a study protocol for a randomized controlled trial**

**Abstract**

To data, the cases of COVID-19 infections are increasing exponentially worldwide. In the absence of approved and efficacious vaccines for managing SARS-CoV-2 infection, there is an urgent need to identify pharmaceutical agents for treatment and prevention of COVID-19. Most of the treatment options available for this disease are based on previous experiences in treating severe acute respiratory syndrome (SARS) and Middle East respiratory syndrome (MERS) coronaviruses. Additionally, new evidence indicates that therapeutic agents with antiviral properties as well as those with anti-inflammatory and immunomodulatory activities may be effective in managing COVID-19 infection. In this regard, it appears that boron-containing compounds and oleoylethanolamide (OEA) supplementation along with routine treatments may have beneficial effects on patients with COVID-19 due to their anti-inflammatory, antioxidant, and antiviral effects.

The study will be piloted on 40 patients with COVID-19 whose disease has been proven by a physician and laboratory findings. Patients will be randomly assigned to four groups. The first group (A) will receive two capsules containing 5 mg boron compounds for two weeks. The second group (B) will receive two capsules containing 200 mg oleoylethanolamide for two weeks. The third group (C) will receive two capsules containing 5 mg boron compounds with 200 mg oleoylethanolamide for two weeks, and the fourth group (D) does not receive any additional treatment other than routine treatments. The primary end point of this study is to investigate the recovery rate of clinical symptoms, including fever, dry cough, and fatigue, as well as preclinical features, including complete blood count (CBC), the erythrocyte sedimentation rate (ESR), C-reactive protein (CRP) profiles within two weeks of randomization.

**Background**

Coronavirus disease 2019 (COVID-19), a novel β-coronavirus caused by infection with severe acute respiratory syndrome coronavirus 2 (SARS-CoV-2), was first detected in early December 2019, in China. To data, the cases of COVID-19 infections are increasing exponentially worldwide. Most common symptoms of COVID-19 infected patients are fever, cough, breathing problems, sore throat, unexplained loss of taste or smell, headache, and finally severe respiratory syndrome (3). In the absence of approved and efficacious vaccines for managing SARS-CoV-2 infection, there is an urgent need to identify pharmaceutical agents for treatment and prevention of COVID-19. Most of the treatment options available for this disease are based on previous experiences in treating severe acute respiratory syndrome (SARS) and Middle East respiratory syndrome (MERS) coronaviruses. Additionally, new evidence indicates that therapeutic agents with antiviral properties as well as those with anti-inflammatory and immunomodulatory activities may be effective in managing COVID-19 infection. In this regard, it appears that boron-containing compounds and oleoylethanolamide (OEA) supplementation along with routine treatments may have beneficial effects on patients with COVID-19 due to their anti-inflammatory, antioxidant, and antiviral effects.

In recent years, peroxisome proliferator-activated receptor-alpha (PPAR-α), a ligand-activated transcription factor, has emerged as a useful drug target for the regulation of pathophysiological functions, including inflammation and oxidative stress. Activation of PPAR-α pathway by its agonists has a favorable effect on lipid synthesis and oxidation, inflammatory processes and the expression of immunomodulatory genes. PPARα has been shown to negatively regulate pro-inflammatory and [acute phase response](https://www.sciencedirect.com/topics/medicine-and-dentistry/acute-phase-response) [signaling pathways](https://www.sciencedirect.com/topics/medicine-and-dentistry/signal-transduction). The bioactive lipid oleoylethanolamide (OEA), is an endogenous high-affinity agonist of PPAR-α. Previous studies have demonstrated that OEA is a potent anti-inflammatory and antioxidant compound. To data, OEA exerts anti-inflammatory effects by enhancing PPARα expression and by suppressing the expression of pro-inflammatory factors such as cyclooxygenase-2 (COX-2), interleukin 6 **(**IL-6), IL-1 β, C-reactive protein (CRP), tumor necrosis factor-α (TNF-α), inducible nitric oxide synthase (iNOS), and toll like receptors-4 (TLR4)-mediated nuclear factor kappa B (NF-κB) signaling cascade. OEA can also act as scavenger for reactive oxygen species (ROS) and increase antioxidative enzymes. OEA is derived from the monounsaturated fatty acid, oleic acid (OA). In poliovirus-infected HeLa cells, incorporation of OA into membranes resulted in increased membrane fluidity, making these membranes nonfunctional for poliovirus RNA replication, and blocked viral RNA synthesis. Additionally, OA could strongly protect cells from the cytopathogenic effect induced by poliovirus.

Boron-containing compounds have recently gained growing interest as potential novel therapeutic agents due to a wide range of biological effects, including antibacterial, antifungal, antiviral, and anti-inflammatory activities. The importance of dietary boron in improving antioxidant defence mechanisms (superoxide dismutase, catalase and glutathione peroxidase enzyme activities) thereby ameliorating oxidative stress has been suggested in previous works. Recent studies have demonstrated that boric acid stimulates the release of chemical mediators of inflammation, which are important in host defense against infections. Similarly, in mice stimulated with lipopolysaccharide (LPS), administration of borax, a salt of boric acid, induced lymphocyte proliferation and increased the release of inflammatory mediators (cytokines and nitric oxide).

The ability of boron to regulate the inflammatory responses has also been reported in human. In the areas of the world where boron intakes were 3 to 10 mg/day, the estimated incidence of arthritis, a condition in which one or more of joints are inflamed, ranges from 0 to 10%. While, in areas where the boron intakes were 1.0 mg or less/ day, the estimated incidence of arthritis ranges from 20 to 70%, demonstrating the protective role of boron in inflammatory processes.

Protease inhibitors blocking the replication of coronavirus and are considered as an effective therapeutic target for diseases caused by coronaviruses, including COVID-19. New evidence indicates that boron-based drugs can be considered as viral protease inhibitors. These compounds showed potent enzyme inhibitory activity. For example, bortezomib, the boron-based drug, is the clinically approved proteasome inhibitor with demonstrated antiviral activity. This drug could block severe fever with thrombocytopenia syndrome (SFTSV) infection by affecting virus infectivity, replication, and release. Moreover, bortezomib could significantly inhibit growth of the influenza virus in Madin-Darby canine kidney and human A549 lung cells.

**Methods and design**

**Study design**

The current study is a single-center, randomized, double-blind, placebo-controlled clinical trial with parallel groups.

**Study population**

The inclusion criteria include male and female patients ≥18 years of age, with a confirmed diagnosis of SARS-CoV-2 infection via polymerase chain reaction (PCR) and/or antibody test and patients with written informed consent to participate in this trial. The exclusion criteria include regular use of any other supplement, severe and critical COVID-19 pneumonia, pregnancy and breastfeeding. This study is being conducted at Imam Reza Hospital, Tabriz University of Medical Sciences, Tabriz, Iran

**Sample size**

The calculated total sample size is 40 patients, with 10 patients in each group.

**Randomization and intervention**

After obtaining the informed consent and expressing the objectives of the research, eligible subjects (n=40) will be divided into four equal groups, using the Random allocation software (RAS), with 1:1 allocation ratio. The block randomization will be conducted by an assistant and the intervention allocation will be blinded for both the investigators and participants. Patients are randomly assigned to four groups. The first group (A) will take one capsule containing 5 mg of boron compounds twice a day for two weeks. The second group (B) will take one capsule containing 200 mg oleoylethanolamide twice a day for two weeks. The third group (C) will take one capsule containing 5 mg boron compounds with 200 mg oleoylethanolamide twice a day for two weeks, and the fourth group (D) does not receive any additional treatment other than routine treatments. Boron-containing compounds and oleoylethanolamide capsules will be synthesized at Nutrition Research Center of Tabriz University of Medical Sciences.

The person (completely unrelated to the study) who is responsible for preparing the supplement capsule will be asked to assign a three-digit code to each of the capsules. OEA and boron-containing compounds were synthesized at the Nutrition Research Center, Tabriz University of Medical Sciences, Iran. All capsules will be similar in terms of shape and color.

**Study outcomes**

The primary end point of this study is to investigate the recovery rate of clinical symptoms, including fever, dry cough, and fatigue, as well as preclinical features, including complete blood count (CBC), the erythrocyte sedimentation rate (ESR), C-reactive protein (CRP) profiles within two weeks of randomization.

**Measurements and assessments**

The recovery rate of clinical symptoms, including fever, dry cough, and fatigue will be assessed before and after intervention. Blood samples (5 mL) will be collected following a 12-h overnight fast and centrifuge at 3000 rpm for 5 min to extract serum samples. Preclinical features, including complete blood count (CBC), the erythrocyte sedimentation rate (ESR), and C-reactive protein (CRP) profiles will be assessed before and after intervention.

**Statistical analysis**

Data entry, coding, security, and storage will be checked. Statistical analysis of all data will be done by SPSS statistical software (SPSS Inc., Chicago, IL, USA, version 23) and P values less than 0.05 will be considered statistically significant. The Kolmogorov-Smirnov test will be used for assessing the normality of the data. Quantitative and qualitative variables will be presented as mean (standard deviation) or median (25th–75th percentile), and frequency (percentage), respectively. Non-parametric, Wilcoxon, and Pearson's correlation coefficient statistical tests as well as t-test will be used. Analysis of covariance (ANCOVA) will be applied to adjust the effects of confounding factors.

**References**

1. Schoeman D, Fielding BC. Coronavirus envelope protein: current knowledge. Virology journal. 2019;16(1):69.

2. Zhu N, Zhang D, Wang W, Li X, Yang B, Song J, et al. A novel coronavirus from patients with pneumonia in China, 2019. New England Journal of Medicine. 2020.

3. Chen Y, Liu Q, Guo D. Emerging coronaviruses: genome structure, replication, and pathogenesis. Journal of medical virology. 2020.

4. Conti P, Ronconi G, Caraffa A, Gallenga C, Ross R, Frydas I, et al. Induction of pro-inflammatory cytokines (IL-1 and IL-6) and lung inflammation by COVID-19: anti-inflammatory strategies. Journal of biological regulators and homeostatic agents. 2020;34(2).

5. Shini S, Sultan A, Bryden WL. Selenium biochemistry and bioavailability: implications for animal agriculture. Agriculture. 2015;5(4):1277-88.

6. Suhajda A, Hegoczki J, Janzso B, Pais I, Vereczkey G. Preparation of selenium yeasts I. Preparation of selenium-enriched Saccharomyces cerevisiae. Journal of Trace Elements in Medicine and Biology. 2000;14(1):43-7.

7. Bowen KJ, Kris-Etherton PM, Shearer GC, West SG, Reddivari L, Jones PJH. Oleic acid-derived oleoylethanolamide: A nutritional science perspective. Progress in lipid research. 2017; 67:1-15.

8. Tutunchi H, Ostadrahimi A, Saghafi-Asl M, Maleki V. The effects of oleoylethanolamide, an endogenous PPAR-alpha agonist, on risk factors for NAFLD: A systematic review. Obesity reviews: an official journal of the International Association for the Study of Obesity. 2019;20(7):1057-69.

9. Sayd A, Anton M, Alen F, Caso JR, Pavon J, Leza JC, et al. Systemic administration of oleoylethanolamide protects from neuroinflammation and anhedonia induced by LPS in rats. The international journal of neuropsychopharmacology. 2014;18(6).

10. Yang L, Guo H, Li Y, Meng X, Yan L, Dan Z, et al. Oleoylethanolamide exerts anti-inflammatory effects on LPS-induced THP-1 cells by enhancing PPARalpha signaling and inhibiting the NF-kappaB and ERK1/2/AP-1/STAT3 pathways. Scientific reports. 2016; 6:34611.

11. Payahoo L, Khajebishak Y, Asghari Jafarabadi M, Ostadrahimi A. Oleoylethanolamide Supplementation Reduces Inflammation and Oxidative Stress in Obese People: A Clinical Trial. Advanced pharmaceutical bulletin. 2018;8(3):479-87.

12. Piomelli D. A fatty gut feeling. Trends in endocrinology and metabolism: TEM. 2013;24(7):332-41.

13. Gross B, Pawlak M, Lefebvre P, Staels B. PPARs in obesity-induced T2DM, dyslipidaemia and NAFLD. Nature reviews Endocrinology. 2017;13(1):36-49.

14. Ghaffari S RN, Tutunchi H, Ostadrahimi A, Pouraghaei M, Kafil B. Oleoylethanolamide, a bioactive lipid amide, as a promising treatment strategy for coronavirus/covid-19. Archives of Medical Research 2020;51(5):464-67.

15. Soriano-Ursúa MA, Das BC, Trujillo-Ferrara JG. Boron-containing compounds: chemico-biological properties and expanding medicinal potential in prevention, diagnosis and therapy. Expert opinion on therapeutic patents. 2014;24(5):485-500.

16. Ban HSN, H. Boron-Based Drug Design. Chem Rec. 2015; 15:616-35.

17. Fernandes GFSD, W.A.; Dos Santos, J.L; Boron in Drug Design: Recent Advances in the Development of New Therapeutic Agents. Eur J Med Chem. 2019; 179:791–804.

18. Travers RL RG, Newnham RE; Boron and arthritis: the results of a double-blind pilot study. Journal of Nutritional Medicine 1990;1(2):127-32.

19. Nikkhah S DM, Naghii MR, Zaeri F, Taheri SM. Effects of boron supplementation on the severity and duration of pain in primary dysmenorrhea. Complementary therapies in clinical practice. 2015;21(2):79-83.

20. Ghosh AKX, Z.; Kovela, S.; Robinson, W.L.; Johnson, M.E.; Kneller, D.W.; Wang, Y.F.; Aoki, M.; Takamatsu, Y.; Weber ITM, H. Potent HIV-1 Protease Inhibitors Containing Carboxylic and Boronic Acids: Effect on Enzyme Inhibition and Antiviral Activity and Protein-Ligand X-Ray Structural Studies. ChemMedChem. 2019; 14:1863-72.

21. Windsor IWP, M.J.; Lukesh, J.C.; Gold, B.; Forest, K.T.; Raines, R.T. Sub-Picomolar Inhibition of HIV-1 Protease with a Boronic Acid. J Am Chem Soc. 2018; 140:14015-8.

22. Nitsche CZ, L.; Weigel, L.F.; Schilz, J.; Graf, D.; Bartenschlager, R.; Hilgenfeld, R.; Klein, C.D. Peptide-Boronic Acid Inhibitors of Flaviviral Proteases: Medicinal Chemistry and Structural Biology. J Med Chem. 2017; 60:511-6.

23. Baker SJ, Sanders V, Akama T, Bellinger-Kawahara C, Freund Y, Maples KR, et al. Boron-containing small molecules as anti-inflammatory agents. Google Patents; 2012.

24. Liu SL, H.; Zhang, K.; Li, X.; Duan, Y.; Wang, Z.; Wang, T. Proteasome Inhibitor PS-341 Effectively Blocks Infection by the Severe Fever with Thrombocytopenia Syndrome Virus. Virol Sin. 2019;34(5):572-82.

25. Shahiduzzaman ME, P.; Xin, G.; Coombs, K.M. Proteasomal Serine Hydrolases Are Up-Regulated by and Required for Influenza Virus Infection. J Proteome Res. 2014;13(5):2223-38.

26. Liu WZ, H.-L.; Duan, Y. Effective Chemicals against Novel Coronavirus (COVID-19) in China. Current Topics in Medicinal Chemistry. 2020.

27. Bhasker TV, Gowda N, Mondal S, Krishnamoorthy P, Pal D, Mor A, et al. Boron influences immune and antioxidant responses by modulating hepatic superoxide dismutase activity under calcium deficit abiotic stress in Wistar rats. Journal of Trace Elements in Medicine and Biology. 2016; 36:73-9.
